# Assessing for comorbidities, determinants and disability during TB treatment

**DOI:** 10.5588/ijtldopen.23.0441

**Published:** 2024-02-01

**Authors:** K. Viney, A. Baddeley, E. Jaramillo, M. Calvi, A. Carlqvist, F. Mavhunga

**Affiliations:** Global Tuberculosis Programme, World Health Organization, Geneve, Switzerland

Dear Editor,

We read with great interest the Editorial by Harries et al. on why TB programmes should assess for comorbidities, determinants, impairments and disabilities during and after TB disease.^1^ We congratulate the authors for highlighting these important issues, for describing some of the practical steps that TB programmes can take and for sharing their initial findings on related implementation research. Harries et al. present clear reasons why comorbidities and TB-associated impairments and disabilities during and post-TB disease should be addressed by TB programmes and the primary healthcare services. The interventions to address these issues can improve treatment outcomes, reduce deaths and relapse, improve mental health, health-related quality of life and social outcomes, and reduce costs.^2^

An estimated 46% of global TB episodes are attributed to five health-related risk factors: diabetes, HIV, tobacco smoking, undernutrition and disorders due to alcohol use.^3^ A further 42% of people with TB are estimated to have mental health conditions.^4^ In addition, one in four people will live with TB-associated disabilities once discharged from TB treatment due to impairments related to the disease or its treatment, mental health conditions and pulmonary disorders being the most prevalent conditions.^4^ The WHO End TB Strategy^5^ and the United Nations High-Level Meeting commitment on the fight against TB^6^ emphasise the need for comprehensive people-centred care for people with TB and comorbidities, as well as for people at risk of poor treatment outcomes or social exclusion, such as TB-associated impairment and disability during and post-TB disease. Over the years, the WHO has issued guidance on interventions to address TB and comorbidities,^7–9^ and in 2023 published the first ever policy brief on TB-associated disability.^10^

Although the widespread implementation of collaborative TB-HIV activities has saved an estimated 9.4 million lives between 2005 and 2021, uptake of guidance for other comorbidities has been variable. Even less frequently implemented has been systematic assessment of TB-associated impairment and disabilities during or following TB disease.^11,12^ To address this, the WHO issued the ‘Framework for collaborative action on TB and comorbidities’,^2^ which aims to enhance the response to TB and comorbidities by establishing and strengthening collaboration across health programmes and across sectors for delivering people-centred services for TB and comorbidities as part of Universal Health Coverage.^2^ The Framework is complementary to, and should be used in conjunction with, WHO guidelines and operational handbooks on TB and key comorbidities and the policy brief on TB-associated disability. These documents can inform the development of national TB strategic plans and strategic plans for key comorbidities, and can help to inform surveillance, monitoring and evaluation activities, as well as implementation research on TB, comorbidities and TB-associated disability.

Harries et al.^1^ outline some clear actions that TB programmes can take to assess and manage comorbidities and TB-associated impairments and disabilities among people affected by TB during and post TB disease. A complete list of WHO-recommended interventions for TB and the key comorbidities is provided in the Framework (see Table).^2^ In addition, the WHO’s new policy brief on TB-associated disability outlines seven key approaches that Ministries of Health can take to improve health and social outcomes for people with TB-associated disability.^10^ Generating much needed data and evidence, including through implementation research is one such activity, and the research described by Harries et al.^1^ is one example. We look forward to seeing the results of additional research studies in this area, which can inform national programming and global policy development. We also encourage ministries of health to analyse their TB surveillance data to determine the burden of key comorbidities and TB-associated impairments and disabilities to inform future interventions and to improve patient care.

**Table.** Health-related risk factors and TB comorbidities, with related interventions recommended in current WHO guidelines.

	Health-related risk factors for TB			Comorbidities associated with poorer TB treatment outcomes	
	Interventions to reduce the burden of TB among people with comorbidities and health-related risk factors			Interventions to reduce the burden of comorbidities among people with TB	
	Find and treat TB	TB preventive treatment	Infection prevention and control	Find and treat comorbidities	Counsel on and prevent comorbidities
Key drivers of TB					
Diabetes	Y	N	Y	Y	N
Disorders due to alcohol use	Y	N	Y	Y	Y
HIV	Y	Y	Y	Y	Y
Smoking	N	N	Y	Y	Y
Undernutrition	Y	N	Y	Y	Y
Other health-related risk factors and comorbidities					
Disorders due to drug use	Y	Y	Y		
Silica exposure, silicosis	Y	Y	Y		
Viral hepatitis	Y	N	Y		
Other clinical risk factors: treatment with anti-TNF-α,* dialysis, organ or haematological transplantation	Y	Y	Y		
Other comorbidities					
COVID-19				Y	N
Mental disorders				Y	Y
Viral hepatitis				Y	Y

* Currently, there is no recommendation on TB screening for people receiving anti-TNF-α treatment; however, treatment of TB infection is recommended.

Y = recommendation exists; N = no recommendation exists currently; TNF-α = tumour necrosis factor alpha.
